# Principles for ethical research in the Himalayas: Decolonising research ethics across the disciplines

**DOI:** 10.1057/s41599-026-06826-8

**Published:** 2026-03-04

**Authors:** Ishfaq Hussain Malik

**Affiliations:** School of Geography, University of Leeds, Leeds, United Kingdom

**Keywords:** Geography, Environmental studies

## Abstract

Research in the Himalayan region has long been shaped by uneven power relations, driven by extractive academic practices, global conservation agendas, and development interventions. Although often unintentional, these approaches have tended to prioritise data collection over reciprocal relationships, producing knowledge that benefits external actors while silencing or marginalising local perspectives and communities. In this paper, I propose a framework for ethical research that centres the agency, knowledge systems, and material realities of Himalayan communities and ecologies. Drawing from community-based and decolonial methodologies, this framework identifies six ethical principles designed to guide researchers in establishing collaborative, respectful, and non-extractive relationships with local peoples and ecologies. These principles include: (1) community collaboration from the outset, (2) Free, Prior, and Informed Consent, (3) knowledge co-production, (4) protection of situated cultural and ecological knowledges, (5) benefit sharing and reciprocity, and (6) long-term commitment and accountability. These principles extend beyond the social sciences, humanities and arts, requiring that all researchers, including those in STEM, environmental monitoring and natural sciences, address how their work risks reproducing colonial and epistemic asymmetries of power through extractive data practices and surveillance, including instrument deployment and sample collection, which can be perceived as acts of academic or institutional territorial claim-making. The paper also discusses the challenges and considerations required for operationalising ethical research principles. By focusing on local authority, lived ecological conditions, and knowledge sovereignty, this paper presents an ethical model for research that challenges extractivism and centres relational responsibility. It urges scholars, institutions, and funders to acknowledge the contested terrains of knowledge and power in which Himalayan communities are embedded—and to respond accordingly with care, humility, and commitment.

## Introduction

The Himalayan region, often portrayed in global imaginaries as a pristine wilderness or spiritual sanctuary, has long served as a frontier of extractive curiosity—an object of study for anthropologists, ecologists, conservationists, and development planners. From high-altitude climate monitoring to ethnographic encounters in remote villages, the Himalayas have been repeatedly subjected to research practices that privilege data over dialogue, observation over reciprocity, and publication over presence. Behind the aesthetics of fieldwork lies a history of epistemic extraction: knowledge taken, codified, and circulated within distant institutions, while the communities who animate this knowledge are left out of authorship, agency, and benefit. These research paradigms, often framed as neutral or benevolent, are deeply embedded in longer colonial trajectories of control and categorisation (Held 2019). They rely on assumptions of objective expertise, universal science, and methodological detachment—assumptions that erase local cosmologies, geographies, and ecological stewardship practices rooted in centuries of lived experience (Yü and Maaker 2021; Dionne 2005). In the Himalayas, where land is not a resource but a relative, and where knowledge is culturally grounded, embodied, and intergenerational (Chakraborty 2018; Guneratne 2010), the application of extractive research logics becomes an act of dispossession. It severs the sacred from the scholarly, and the relational from the rational.

Globally, scholars and communities have critiqued dominant research paradigms that perpetuate epistemic violence (Brunner 2021; Malik et al. 2025a, 2025b; Ndlovu and Ndlovu-Gatsheni 2024). Rooted in Euro-Western ontologies and extractivist logics, these frameworks often treat knowledge as a commodity to be extracted, owned, and interpreted by external actors, thereby excluding the voices, consent, and sovereignty of Indigenous and local communities (Smith 2021, Tuck 2009; Wilson 2020). Within the Himalayan region, similar experiences are creating a recurring pattern of extractive research. This is evident in the physical and environmental sciences, where top-down data collection has at times blamed local communities for degradation while ignoring their land-management expertise and marginalisation of local environmental history (Ives and Messerli 2003; Forsyth 1996).

In fields such as ethnomedicine, this extraction manifests as biopiracy, where Indigenous pharmacological knowledge is commodified for global markets without benefit-sharing (Shiva 2016). Critiques within anthropology and development studies have highlighted how ‘helicopter’ research practices frequently marginalise local political voices in favour of external agendas (Shrestha 1997; Gergan 2017). The result is a frequent absence of genuine community participation and the continued commodification of ecological and cultural knowledge.

Research in the Himalayas is further complicated by overlapping interests from development agencies, conservation NGOs, climate scientists, national security institutions, and state-led policy and planning (Rana et al. 2022; Mishra et al. 2019). Each actor brings its own agenda, requirements, and bureaucratic procedures (Sherman et al. 2012), often justified under the guise of development, sustainability, resilience, or modernisation. These projects tend to abstract local life into datasets, downplaying the lived experiences of people whose lands are being mapped, monitored, and transformed. Himalayan communities contend with intersecting challenges, including climate change, geopolitical tension, ecological degradation, poverty, unemployment, extractive development projects, and the erosion of cultural heritage (Malik and Ford 2025a; Chahal et al. 2024; Li and Shapiro 2020). In such a context, the ethics of research cannot be confined to bureaucratic protocols or individual consent. Instead, they must be grounded in a broader understanding of entangled relations between knowledge, power, place, and community sovereignty.

While much critique has been directed toward Euro-Western academic and development institutions, it is equally important to recognise that mainstream institutions and research bodies within South Asia—particularly those affiliated with mainstream universities, environmental think tanks, and Himalayan development agencies—have often perpetuated similar extractive logics. These institutions, though geographically closer, are not necessarily epistemically aligned with the communities they study; they frequently operate through top-down agendas, universalist science, and policy-driven imperatives that sideline local worldviews. Likewise, mainstream research institutions and infrastructure-led projects in the Trans-Himalayan region have raised concerns regarding data extraction, cultural marginalisation, and the securitisation of knowledge (Panda 2024; Davis et al. 2021).

The extraction projects in the Himalayas by the countries inhabiting the region are causing what is known as ‘slow violence’ by Nixon (2011) on the region’s ecologies and cultures, representing a threat to the communities, ecologies, and sociocultural wellbeing (Davis et al. 2021). These examples underscore that extractivism is not the exclusive domain of colonial or foreign actors, but a structural feature of research paradigms embedded in state, academic, and technocratic institutions across the region.

Decolonial methodologies aim to restructure the foundational assumptions of research itself. Walter Mignolo (2007) and Aníbal Quijano (2000) propose a ‘delinking’ from colonial matrices of power and knowledge, advocating instead for epistemic plurality and the validation of Indigenous and cultural worldviews. Decolonial ethics stress relational accountability, community ownership of data, and the right of communities to refuse participation (Lenette 2022; O’Connell 2016; Tuck and Yang 2012). Community-based participatory research, participatory action research, and Indigenous research methodologies have emerged as counter-models to dominant paradigms (Smith 2021; Beveridge et al. 2021; Zavaleta-Cortijo et al. 2024). These approaches centre collaboration, co-learning, and relational accountability, while resisting the institutional norms that privilege expert authority over lived experiences and knowledge (Ford et al. 2024; Köppen et al. 2022; Malik and Ford 2025b). While scholars such as Gohain (2020) have demonstrated how grounded ethnographic methods can embody these principles in the Himalayas, widespread operationalisation remains constrained within dominant research models. This is often due to linguistic, political, and infrastructural barriers, the institutional inertia of academic and neoliberal-driven research frameworks, and the performative adoption of ethical language. Co-development of ideas has become ubiquitous in research rhetoric, particularly within the physical sciences, yet it is often invoked without meaningful engagement, genuineness or practice, leaving underlying power hierarchies and extractive methods intact.

Dominant modes of knowledge production also operate within uneven terrains of access and authority (Lund and Öztok 2025). Academic research is mediated by funding priorities, linguistic hierarchies, institutional affiliations, and the geopolitics of borderland governance (Mamadouh and Dorfman 2023; Castanho 2023). These structural forces often render communities legible only through developmental or environmental registers (Kolosov 2023; Natarajan et al. 2022). In doing so, research can reinforce the very inequalities it claims to address, reproducing hegemonic representations while obscuring resistance, resilience, and refusal (Glynn and Cupples 2024; Theriault and Mowatt 2024).

What is needed, then, is not merely a revision of ethical protocols but a rethinking of research itself: a shift from extractive to relational, from representational to co-constitutive, and from disciplinary to dialogical. Ethical research in the Himalayas demands attention not only to individual consent but to collective sovereignty; not only to human subjects but to more-than-human entanglements; not only to what knowledge is produced but how, for whom, and with what consequences. This paper proposes a framework of ethical principles rooted in community-centred engagement, aimed at transforming research from a colonial residue into a practice of shared responsibility and situated care.

A growing body of scholarship across the social sciences—from constructivist grounded theory (Charmaz 2006) to critical international relations (Bleiker 2000; Harman 2019), decolonial anthropology and ethnography of resource politics (Kikon 2019; 2017), and the material ethics of Himalayan objects (Martin 2017)—has stressed the imperative of methodological reflexivity, positionality, and relational ethics. These theoretical frameworks highlight that knowledge is co-constituted, not merely extracted, demanding that researchers account for their subjective role and ethical presence in the research encounter. While these approaches provide the philosophical foundation for non-extractive research, their effective application requires situated adaptation. The Himalayan region presents a unique nexus of challenges—including entrenched geopolitical borders, diverse and often contested Indigenous knowledge systems, and historical extractive practices across physical and social sciences (Sharma and Murton 2025; Godara et al. 2024)—which necessitate a highly specific framework for implementation. The subsequent principles, therefore, are designed not to reinvent ethical theory, but to articulate a robust, context-specific methodology that translates these existing critical imperatives into tangible practice for the Himalayan empirical setting.

This paper is guided by the central research question: *How can ethical research practices be conceptualised and implemented in the Himalayan context in ways that are grounded in community values, epistemologies, and priorities, rather than extractive or externally imposed paradigms?* In addressing this question, the study draws on lived experiences, Indigenous methodological frameworks, and a critical reading of existing ethical challenges to propose six foundational principles. Implementing these principles, however, requires more than methodological tools—it demands deep contextual understanding of the region’s complex social, political, and ecological histories. Such depth is often overlooked or deemed too difficult by many researchers, contributing to the persistence of surface-level ethical engagements rather than genuine transformation.

This paper aims to i) develop a Himalayan-specific ethical framework for conducting research—one that is decolonial, participatory, and rooted in community-defined epistemic values; ii) propose six key principles for ethically engaging with the Himalayas, emphasising relational accountability, situated knowledges, and local authority over data and interpretation; iii) support ethical research that does not reproduce extractive logics but rather supports knowledge sovereignty, community needs and priorities, and nourishes local autonomy and ecological futures; and iv) critically deconstruct the underlying structural biases in Himalayan research by identifying how ontological bias and epistemic privilege operate as mechanisms that reproduce colonial knowledge asymmetries and sustain extractive research logics in practice.

## Methodological and theoretical orientation

This paper is fundamentally informed by two complementary methodological approaches: decolonial and participatory frameworks and the literature on usable science and actionable knowledge (Smith 2021; Mach et al. 2020; Omodan 2025a; Lemos et al. 2018). These frameworks together foreground key principles for ethical engagement proposed in this study. This approach emphasises that researchers and knowledge users must meaningfully interact to co-create knowledge that is actionable in decision-making and helps in understanding what works in what contexts and how to avoid potentially undesirable outcomes (Bradbury 2015; Jull et al. 2017; Malik et al. 2025c; Kovach 2021; Datta 2018). The study employs a conceptual and normative methodology based on systematic literature review and critical discourse analysis to rigorously formulate six principles through a synthesis of existing literature, a critical review of research policy, and theoretical engagement with justice concepts.

The ethical principles proposed here are not universal templates, but contextual guidelines rooted in Indigenous and subaltern demands for autonomy, justice, and recognition. They are drawn from engagement with both scholarly literature and lived observations of research practices in the Himalayan region. The emphasis is on developing research ethics that are place-sensitive, responsive to local governance structures, and grounded in a critique of power asymmetries embedded in funding, language, and academic authority. This is not a value-neutral exercise: it requires researchers to confront their own positionality and to engage with research as a political act, deeply implicated in systems of dispossession and resistance.

This framework draws upon i) decolonial theory and community collaboration (Mignolo 2007; Quijano 2000; Pearce et al. 2009) that calls for epistemic disobedience and delinking from Eurocentric knowledge hierarchies and active involvement and collaboration with community members and local, regional and national organisations that use this research for policy-making; ii) Indigenous methodologies (Smith 2021; Wilson 2020; Kovach 2021; Latulippe and Klenk 2020) that emphasise relational accountability, co-responsibility, and seeking Indigenous knowledge sovereignty to inform environmental decision-making, implying that Indigenous Peoples are stakeholders with rights and responsibilities regarding their knowledge systems and lands; and iii) CARE (Collective Benefit, Authority to Control, Responsibility, and Ethics) Principles for Indigenous Data Governance (Carroll et al. 2023; Barsness et al. 2023) that guide responsible stewardship and collective control over Indigenous knowledge. This methodology advances the principle that Himalayan communities and ecologies are not research subjects—they are rights-bearing knowledge holders. These frameworks go beyond ‘do no harm’ and advocate for restorative, reciprocal, and sovereign research relationships.

## Situating the Himalayas: ecologies, communities, and research agendas

The Himalayas are the tallest mountain ranges in the world, often known as the third pole due to their large ice cover on the earth outside the polar regions, and constitute one of the world’s most ecologically diverse regions (Singh and Pusalkar 2020; Sati 2020). Himalayan rivers are among the most important hydrological and cultural systems in the world, originating in the high glaciated peaks and ultimately sustaining over a billion people downstream (Qazi et al. 2020; Lepcha et al. 2021). The human geography of the Himalayas is characterised by a complex interplay of diverse ethnic groups, settlement patterns, and cultural and economic activities (Ghimire 2020; Ahmad 2009; Guneratne 2010). They are home to a variety of cultures who have adapted to the environment through subsistence agriculture, nomadic herding, and traditional crafts, as well as urban settings (Joshi et al. 2020; Sharma et al. 2017).

The Himalayas are home to hundreds of Indigenous, semi-nomadic, and long-established communities – such as Lepchas, Bhutias, Sherpas, Bhotias, Brokpas, Monpas, Baltis, Tamang, Gurung, Magar, Rai, Ladakhis, Paharis, Gujjars, Bakerwals, Kashmiris – among many others with their own languages, oral traditions, spiritual systems, and land-based livelihoods (Sharma and Sood 2017; Malik 2022a, 2022b; Bhasin 2011; Timothy and Nyaupane 2022). In places like Zanskar and Mustang, Buddhist cosmology organises relationships with land and water; in Arunachal Pradesh, animist and Donyi-Polo practices govern forest and river stewardship; and in Kumaon and Garhwal, sacred groves and pilgrimage routes such as Kedarnath and Hemkund Sahib illustrate how spiritual and ecological worlds are entangled (Kumar et al. 2020; Modi 2022; Manocha and Pasupuleti 2024; Jamatia 2023). Himalayan communities have developed unique cultural adaptations to the challenging environment, including traditional clothing, housing, and social structures (Pandey et al. 2018; Guneratne 2010).

Contemporary research in the Himalayas is driven by a wide range of disciplinary and policy concerns. Key themes include climate change and adaptation, glacial retreat, disaster risk (especially related to floods, landslides, earthquakes, and Glacial Lake Outburst Floods, GLOFs), biodiversity conservation, infrastructure development, hydropower expansion, cross-border militarisation, sustainable tourism, and documentation of intangible cultural heritage (Nyaupane 2022; Malik and Ford 2024; Yadav and Singh 2024; Malik and Ford 2025a; Sharma and Pathak 2024; Malik and Hashmi 2021). These research agendas are linked to international development initiatives, national security concerns, or global conservation efforts—often framed in technocratic or ecological terms that obscure the lived experiences of local communities.

## Positionality statement

As a Kashmiri researcher from Anantnag, Kashmir—situated within the Himalayan region, this research is not a detached academic pursuit but a deeply personal, situated, relational, and ethical commitment. It emerges from lived experiences, regional entanglements, and an acute awareness of the power dynamics that shape knowledge production in and about the Himalayas. I write from within the landscapes, cultures, and communities that are often framed as research sites by external observers. This rootedness offers me an understanding of the complex entanglements between land, livelihood, ecology, and governance that characterise everyday life in the region. I bring to this research an embedded understanding of the socio-cultural and ecological realities of the Himalayan region. My positionality and over a decade of fieldwork experience provide both an embedded understanding of local lifeworld and a reflexive awareness of the responsibilities involved in engaging with, and amplifying, community knowledges in ethically grounded ways. I am embedded in the political ecologies of extraction and conservation, of cultural landscapes and securitised borders. While my embeddedness affords me linguistic, cultural, and ecological familiarity, I remain mindful that proximity does not absolve power. I know firsthand how development agendas, environmental projects, and even well-meaning scholarship can disrupt local systems of meaning, redistribute power, and reconfigure access to land and resources. These experiences inform my commitment to a research praxis that resists abstraction, recentres community voices, and acknowledges the material and political consequences of knowledge production. This research is guided by an ethic of responsibility: to remain accountable to the communities that hold the knowledge, stories, and cosmologies I seek to engage with; to prioritise relationality over representation; and to challenge the hierarchies—colonial, disciplinary, and institutional—that often define who is allowed to know and who is allowed to speak.

## Principles for ethical research in the Himalayas

**Fig. 1:**
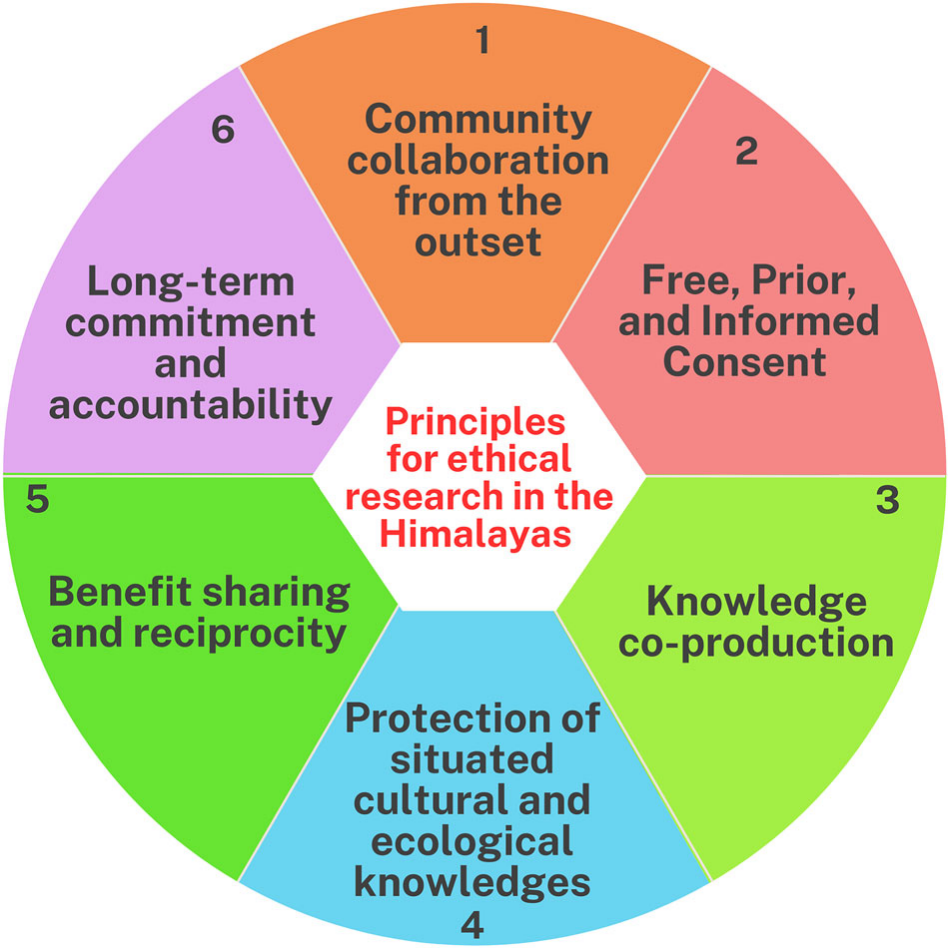
Principles for ethical research in the Himalayas.

The six principles presented in this section (Fig. 1 and Table 1) establish standards for research conduct, aiming to provide a framework that can transform ethical practice from a mere checklist of institutional compliance into a continuous, political commitment. This framework is founded on the principle that ethical responsibility requires active accountability and deep engagement with community sovereignty across all disciplines. The principles, schematically represented below, organise the necessary actions across the full lifecycle of a research project—from co-designed inception to post-project continuity. These standards are essential for ensuring that all scientific endeavours actively advance justice and support Himalayan communities and ecologies. These principles are not intended as rigid rules, but as generative commitments to guide researchers toward more just, accountable, and collaborative practices.

**Table 1:** Principles for ethical research in the Himalayas, outlining six core principles and related actions for decolonial, community-centred, and accountable research engagement.

Principle	Core concept	Key actions	Governance	Transparency
Community collaboration from the outset	Equitable relationships and co-leadership from the outset	- Co-develop research goals and methods - Engage community members, councils, elders, women’s groups, and youth - Build from relational trust, kinship norms, and hospitality ethics	- Community ethics boards with rights-holders and elders - Recognise local governance for project approval	- Disclose funding, affiliations, and intended outcomes - Maintain transparency from early scoping onward
Free, Prior, and Informed Consent	Respect for community autonomy and decision-making authority	- Secure voluntary, timely, and informed consent - Use culturally appropriate and multilingual formats (oral, visual, ritual) - Consent is ongoing, revocable, and collective	- Collaborative consent protocols updated over time - Honour local leadership and kinship-based decision structures	- Communicate research intentions clearly in advance - Respect the right to refuse or withdraw at any stage
Knowledge co-production	Shared authorship and epistemological parity	- Frame questions, methods, and findings jointly - Use culturally grounded tools (story circles, walking interviews, exhibitions) - Value oral, embodied, and ritual knowledges as valid epistemologies	- Shared interpretation and co-authorship in all research phases - Community review of findings before publication	- Ethical dissemination through community report-backs - Acknowledge community contributions and intellectual rights
Protection of situated cultural and ecological knowledges	Respect for sacred, restricted, and intergenerational knowledges	- Avoid extraction of sacred or restricted information - Align with the Nagoya Protocol and CARE principles - Support Indigenous data sovereignty and localisation	- Allow communities to decide what can be recorded, shared, or withheld - Apply community protocols over institutional data mandates	- Disclose intended data use, storage, and access permissions - Avoid uploading sensitive data to open-access repositories without consent
Benefit sharing and reciprocity	Research must actively benefit communities, not just avoid harm	- Provide fair compensation, honorarium, training, and tools for capacity sharing - Support ecological, cultural, or political goals identified by the community - Enable community-led monitoring	- Let communities define what counts as a benefit - Build long-term partnerships beyond the project cycle	- Be transparent about who benefits from the research - Clarify how outputs will be used (policy, advocacy, etc.)
Long-term commitment and accountability	Ethical responsibility continues beyond fieldwork and publication	- Remain accessible for post-research communication - Translate findings into usable local outputs - Co-develop future projects with community input	- Conduct community ethics audits or host public report-backs - Allow for community critique and modify outputs accordingly	- Report on how findings are used externally - Commit to relational ethics grounded in care, respect, and solidarity

### Community collaboration from the outset

Ethical research begins before data collection—with the formation of equitable and reciprocal relationships. Researchers must involve communities in identifying research goals, methodologies, and potential risks from the very beginning (Fielding-Miller et al. 2022; Banks 2022; Wood and Kahts-Kramer 2023). This means conducting scoping visits to engage with community members, local councils, elders, women’s groups, youth, adults, and leaders to co-develop research designs that reflect community priorities and epistemologies. Community collaboration must include transparency about funding, institutional affiliations, and intended outputs. Without this transparency, research reproduces asymmetries of access and interpretation. Ethical research collaboration must be rooted not only in process but in epistemological reorientation. Drawing from Indigenous and decolonial research traditions, collaboration must reject extractive paradigms that treat local knowledge as a means to an academic end (Smith 2021; Chilisa 2019).

Ethical research in the Himalayas must begin with a fundamental reorientation of authority—from the academic researcher to the community. Too often, research is initiated within institutions, with community members engaged only at the stage of data collection or validation. This practice reinforces power asymmetries and perpetuates extractive knowledge systems. Instead, ethical research must begin with community co-identification of research priorities, ensuring that the concerns, questions, and aspirations of local people drive the research agenda (Aragon 2024; Hillyer 2023; Babchuk et al. 2020). This commitment to shared authority is central to the tradition of participatory action research and its demand for democratic knowledge creation (Fals-Borda 1987).

Community collaboration involves more than consultation; it demands genuine partnership and shared decision-making. Engaging with community members and traditional knowledge holders in the planning phase enables culturally grounded guidance. This participatory approach aligns with Indigenous research frameworks that prioritise relational accountability—the understanding that knowledge is co-created through trust, reciprocity, and sustained relationships (Wilson 2020; Nutton et al. 2020). It is rooted in the methodological imperative of establishing trust and ethical presence before research commences (Kovach 2021). In such paradigms, research is a practice of care, not extraction.

Community members should be involved into all phases of research—including funding proposals, fieldwork logistics, and interpretation of results. This ensures not only procedural ethics but also substantive ethical alignment with community values, cosmologies, and needs. In the Himalayan context, this includes building partnerships that are sensitive to local systems of governance and inclusion. For instance, researchers must engage with village councils, tribal bodies, monastic institutions, or women’s federations, depending on the cultural and political landscape. Such contextualisation is essential to ensure that power is not simply redistributed but reshaped in ways that reflect community priorities.

While this paper advocates for research frameworks that centre community engagement and sovereignty, it is also important to recognise that ‘the community’ is not always a homogenous or unified entity, differing in cultural entities such as ideas, knowledge, or attitudes, and the complexity of social structures (Heydari Fard 2024; Khmelnitsky and Kagan 2013). Social relations within Himalayan communities are shaped by caste, gender, class, age, ethnicity, religious authority, and land ownership, which have been formulated over decades to centuries. These structures influence whose voices are heard, who has access to decision-making, and who is marginalised in knowledge processes (Das et al. 2024; Kalyanwala 2025). Importantly, collaboration must contend with these internal community dynamics. Ethical collaboration requires identifying and amplifying the perspectives of those traditionally excluded—especially women, communities at the bottom of the caste hierarchy, and migratory or pastoral groups—who may not hold formal authority, but whose lived knowledge is vital (Thapa et al. 2021; Malik et al. 2024; Chakraborty et al. 2023).

The intersecting social fault lines often determine the ontological biases and epistemic responses of researchers, resulting in research practices that reproduce colonial, statist, and intra-regional hierarchies. The research that results from this unevenness frequently privileges the knowledge of dominant landowning or caste groups, while rendering the experiences of marginalised groups (such as women, lower-caste groups, or nomadic herders) ontologically invisible and epistemically irrelevant (Gupta 1998; Rana 2026; Wani and Malik 2023; Gergan 2014). Without careful attention to these dynamics, participatory research can risk reproducing existing hierarchies rather than dismantling them. An ethics of engagement must therefore be attuned not only to external power asymmetries between researchers and communities, but also to internal differences and silences within communities themselves.

The language of capacity building must be replaced with capacity sharing—a reciprocal process where both researchers and community members learn from one another. Training youth as co-researchers is not only important in data collection but also strengthening intergenerational transmission of ecological knowledge. Creating community ethics boards composed of rights holders, elders, women’s collectives, and youth groups, and building consent through ritual dialogue, storytelling, and long-term presence should ensure that research is grounded in community values and traditions. Importantly, participation in such ethics boards must be ethically supported through appropriate forms of recognition, including fair compensation for time, knowledge, and labour. These boards function not merely as advisory structures but as gatekeepers of legitimacy. Similar to ethics boards used in Arctic Indigenous research (Hayward et al. 2021; Held 2020), they can approve project design, oversee budget allocations, and co-author ethical protocols. Such models shift decision-making power toward communities.

Collaboration must include not only participation, but community governance through local ethics boards. Researchers must recognise community-level, or tribal authority structures as legitimate sources of ethical decision-making. When collaboration is approached as relational, political, and epistemic engagement—not just technical inclusion—it becomes a powerful foundation for decolonising research practice. This principle must therefore be understood not as a procedural step, but as a sustained commitment to knowledge justice.

### Free, prior, and informed consent (as a process)

Free, Prior, and Informed Consent (FPIC) is a foundational ethical standard recognised in the United Nations Declaration on the Rights of Indigenous Peoples (UNDRIP 2007). This principle operates as a fundamental assertion of collective sovereignty and is indispensable to decolonial research praxis (Tuck and Yang 2012). It affirms the right of communities to make autonomous decisions about any research, development, or policy that affects them (Strauss 2001; Lomelino 2015; Ignace et al. 2023). In the Himalayan context, this principle is especially vital due to historical experiences of disenfranchisement and marginalisation in research processes (Chakraborty et al. 2023; Weaver et al. 2022).

‘Free’ means that consent is given voluntarily, without coercion or manipulation. ‘Prior’ refers to the timing—consent must be obtained before any research activities begin. ‘Informed’ means that communities must receive accessible, comprehensive information about the goals, methods, risks, and benefits of the research. Informed Consent must be understood as a continuous and situated process, not a one-time signature on a form. This processual understanding challenges the transactional, bureaucratic ethics model often employed by Western institutions (Porsanger 2004).

In decolonial and Indigenous research frameworks, FPIC is not simply a procedural requirement, but a relational and political commitment to self-determination, collective rights, and ethical research governance (Yako 2021; Blagg and Anthony 2019; Loke and Owen 2024). Recent scholarship underscores that FPIC is most effective when operationalised through community-specific consent protocols that reflect local governance systems, cosmologies, and communication norms (Carroll et al. 2020; Schilling-Vacaflor and Flemmer 2020).

Implementing FPIC in Himalayan communities requires local-language communication, culturally appropriate materials (e.g., visual aids, participatory whiteboard videos or storytelling formats), and multiple forms of expression—verbal, written, ritual, or symbolic. Such tools are important for community engagement and relevance, can reinforce environmental knowledge, and potentially encourage behavioural change (Saini et al. 2020). Importantly, communities must have the sovereign right to withdraw from research at any stage, and their decision must be respected without any consequences.

Consent processes should be documented and revised collaboratively to reflect evolving understanding and expectations. This means not only obtaining consent at the beginning but revisiting it throughout the research lifecycle—during publication, dissemination, and future reuse of data (Tynan 2021; Muller et al. 2023). Researchers must acknowledge that decisions regarding environmental governance, resource use, and ecological knowledge are often shaped through collective processes involving diverse actors—such as community members, local councils, kinship groups, women’s collectives, spiritual leaders, and customary institutions. Relying solely on individual consent can obscure these complex social dynamics and overlook the plural, and sometimes contested, forms of authority that govern community knowledge and land relations.

In contexts where knowledge is considered collectively owned, privileging individual signatures may distort the ethical legitimacy of the research (Carpenter et al. 2009). FPIC thus demands attentiveness to who gives consent, on whose behalf, and with what kind of authority. Communities should be given the opportunity to define the terms of refusal and dissent. This includes the ability to veto parts of a project, restrict access to specific knowledge domains, or impose conditions on dissemination. These elements are central to upholding knowledge sovereignty and cultural autonomy (Walter et al. 2021). Climate change adaptation interventions in the Himalayas cannot achieve long-term efficacy or ethical legitimacy without deep, sustained community engagement that recognises local agency and traditional ecological knowledge.

When treated as a living process embedded in relationships, FPIC becomes a vehicle for ethical co-existence—not just compliance. It ensures that research is grounded in local political, cultural, and epistemological realities, affirming the community’s right to say yes, no, or not now, on their own terms.

### Knowledge co-production

Decolonial ethics emphasise that knowledge is not discovered by researchers and transmitted to communities; rather, it is co-produced through situated, dialogic engagement (Smith 2021; Lenette 2022). The commitment to treating local knowledge systems as coequal epistemologies is an important mechanism for enacting epistemic justice (Fricker 2007). Building on a decolonial foundation, co-production of knowledge recognises that research is a dialogic, relational process—not a unidirectional transfer of expertise from academia to ‘the field’ (Smith 2021; Chilisa 2019). In the Himalayas, community knowledge systems are deeply embodied in land and water, rituals, oral traditions and histories, ecological practices, and cosmologies (Chakraborty et al. 2021; Malik and Hashmi 2022). Such knowledge is not supplemental to academic inquiry but foundational to understanding socio-ecological realities in the region.

Knowledge co-production has advanced thinking on how to create usable knowledge that fits decision contexts to address societal challenges (Mach et al. 2020). Co-production is not merely collaborative data collection, but a shared epistemic labour. It involves communities not only in gathering data, but in shaping the very questions, methods, and interpretive frameworks of the research (Norström et al. 2020; Fazey et al. 2014). This approach respects Indigenous and local epistemologies as coequal to scientific paradigms and enables relational accountability (Wilson 2008; Ali and Talbert 2024). Thus, research in the Himalayas must treat local knowledge systems not as data sources but as coequal epistemologies. Ethical research must create space for non-Western epistemologies to shape research design, methods, and meaning.

Colonial patterns in academia are often reinforced when Western institutions claim sole credit for discoveries in the Himalayas, systematically marginalising the essential contributions of local collaborators and Indigenous experts. Decolonising research requires a shift from viewing Indigenous and local knowledge as supplementary to recognising it as foundational, necessitating a dismantling of systems that erase local leadership in favour of dominant academic narratives.

Co-production necessitates shared authorship that extends beyond the final publication to encompass the entire research lifecycle. This ensures that community agency is embedded from the initial conceptualisation to the ultimate application of findings. The act of ‘interpreting data’ should be reframed as interweaving multiple worldviews, where local explanatory models are considered equally valid alongside scientific paradigms. This interweaving is a crucial step in recognising the ontological validity of situated knowledges (Agrawal 1995). Knowledge co-production must explicitly confront the power dynamics inherent in knowledge generation, ensuring that marginalised voices are not merely ‘included’ but actively empowered to critique and redefine the research outcomes (Chambers 1997; Khan et al. 2022).

To facilitate genuine co-production, researchers should employ participatory and culturally appropriate methods such as walking interviews, story circles, participatory mapping, and community-led exhibitions. These methods not only democratise data collection but also validate diverse ways of knowing and expressing, and foster known protective factors that underpin community resilience (Flynn and Ford 2020; Norström et al. 2020; Nunn 2022; MacDonald et al. 2015). The co-production of knowledge should facilitate engaged research that empowers Himalayan environmental justice movements, informs culturally grounded education, and strengthens evidence-based policymaking.

Co-production should extend beyond collaborative data collection to encompass the ethical dissemination and shared governance of knowledge outputs. This includes mechanisms such as community report-backs, co-authored publications, vernacular media, participatory theatre, and visual storytelling—tools that democratise the research process and ensure that findings are culturally resonant, locally meaningful, and actionable. This approach affirms that knowledge belongs not to the academic institution or individual researcher, but to the people and places that co-produced it (Collins et al. 2018; Tracy 2024). Dissemination should not be a unidirectional transfer of results, but a co-curated process shaped by community preferences, linguistic realities, and cultural idioms. For example, knowledge may be shared through wall newspapers, posters, village festivals, or local radio, which might be more impactful than academic articles in remote Himalayan contexts. They must be communicated in the local languages or dialects spoken by the intended audiences, ensuring accessibility, relevance, and cultural intelligibility.

Importantly, knowledge co-production must also address ownership, control, and the right to withhold knowledge. Researchers must adhere to Indigenous data sovereignty principles, such as those articulated in the CARE Framework—which emphasises Collective benefit, Authority to control, Responsibility, and Ethics (Carroll et al. 2020). These principles assert that data derived from collective knowledge must be governed by the communities themselves, aligning with their values and priorities. As such, before publication or external dissemination, communities must be consulted to review, amend, or even retract material that may breach cultural protocols or undermine trust.

This approach upholds a standard in which rigour and relational accountability are co-constitutive, and where research outputs are judged not only by academic merit but by their usefulness in supporting community-defined goals and decision-making structures (Cash et al. 2003). Ethical research is therefore incomplete until the communities involved have validated its process, its findings, and its legacy.

Finally, knowledge co-production should not be romanticised. Communities are not homogeneous, and tensions around who is authorised to represent knowledge—based on caste, class, gender, age, or affiliation—may emerge. Navigating these complexities requires long-term presence, reflexivity, and humility on the part of the researcher.

### Protection of situated cultural and ecological knowledges

Himalayan communities and ecologies are rich in intangible cultural heritage, including oral histories, ritual practices, and knowledge systems concerning medicinal plants, sacred landscapes, water cycles, and seasonal patterns (Chakraborty et al. 2021; Bhatt et al. 2024). This knowledge is deeply cultural and often restricted by ritual boundaries, necessitating an ethical framework for engagement (Tiwari and Sunny 2024; Sharma et al. 2024). It is often highly contextual, culturally embedded, and transmitted intergenerationally (Sood and Dhyani 2024; Kumar and Shoshta 2024). Unethical research can expose such knowledge to commodification, misinterpretation, or exploitation, particularly in fields like ethnobotany, folklore, religion, genetics and genomics, pharmaceutical and cosmetic R&D (bioprospecting), archaeology, linguistics, and Traditional Ecological Knowledge (TEK) studies. In an era of bioprospecting and cultural commodification, protecting local knowledges is a political and ethical imperative that addresses the colonial legacy of knowledge appropriation (Shiva 2016; Braidotti 2013; Pushpangadan et al. 2018; Shree 2023).

To protect cultural knowledge, researchers should recognise and respect cultural boundaries, including sacred, secret, or restricted domains of knowledge that are governed by local protocols. The capacity of a community to withhold or restrict access to certain knowledge is a crucial political act, asserting control over cultural capital and ensuring the long-term survival of specific epistemes (Simmel 1906; Mazzocchi 2022). International frameworks such as the Nagoya Protocol on Access to Genetic Resources and the Fair and Equitable Sharing of Benefits Arising from their Utilisation provide important legal and ethical guidance, affirming the rights of Indigenous and local communities to control access to their traditional knowledge and to negotiate fair benefit-sharing agreements (CBD 2011). Ethical research engagement must align with these principles, ensuring that cultural knowledge is not extracted, commodified, or disclosed without explicit, prior, and informed community consent.

Ethical practice requires asking not only ‘can we share this knowledge?’ but also ‘should we share this?’—a question to be answered by the community, not the researcher. This principle aligns with data sovereignty—the right of communities to control how their knowledge is collected, stored, accessed, and used (Reyes-García et al. 2022; Walter et al. 2021). Digital platforms must be developed with consent using data localisation if needed. In some cases, the most ethical choice is not to collect certain data at all.

Informed by Indigenous data governance frameworks such as the CARE principles, cultural knowledge protection reasserts that research ethics are not merely bureaucratic, but deeply moral and political commitments (Carroll et al. 2023; Tynan 2024; Lefthand-Begay et al. 2024). Data sovereignty means communities must have control over how, where, and whether their knowledge is stored, shared, or cited. Knowledge sovereignty includes the political right to remain silent and to reject the commodification of culture (Khalid et al. 2023).

Data should not be uploaded on open-access platforms without approval from community members as some members might be hesitant or fear of any consequences particularly at politically sensitive places. This focus on community control over dissemination is critical to preventing the epistemic violence inherent in open-access mandates that strip knowledge of its situated political context (D’Ignazio and Klein 2023).

### Benefit sharing and reciprocity

Ethical research must go beyond ‘do no harm’ and seek to actively benefit the communities involved (Fielding et al. 2022; Hammett et al. 2022). This includes both tangible benefits—such as fair compensation, honorarium to participants, hiring local research assistants, transport and other necessary facilities, buying fieldwork kits and other necessary items from local people wherever possible, or infrastructural support—and intangible benefits, such as capacity sharing (e.g., training youth, elders, and women in research), educational exchange, and increased local influence over development policies and co-designed advocacy tools that support community goals. In the Himalayan region, data collected from communities is often monetised, published, or used in policy while those who contributed receive no feedback or gain (Rana et al. 2022; Stevens and Satterfield 2024). Knowledge is extracted for publications that offer no return to the people who provided the insights. This reproduces extractive dynamics in which communities remain data sources, not partners. Instead, benefit-sharing must be a negotiated, community-defined process that reflects local priorities, values, and aspirations. For example, some communities may value heritage protection, watershed governance tools, or youth training programs over conventional ‘capacity-building’ workshops.

Ethical practice demands a model of reciprocity, where communities receive as much as they give. This active commitment to benefit-sharing reframes the research relationship away from extraction and toward an ethic of solidarity and situated care (Haraway 2020; Tronto 2020). Researchers must ask, repeatedly: *Who benefits from this work? And how?* A critical dimension of this principle is fulfilling the political demands of epistemic justice by returning usable and relevant knowledge that supports the community’s self-determination (Whyte 2018). This moves research toward relational accountability (Wilson 2008) and transformative justice, where benefits are shared across temporal, generational, and epistemic lines (Tuck and Yang 2014; TallBear 2014). Such benefit is not a charitable act, but an ethical responsibility that recognises historical asymmetries and redistributes intellectual and material value. Acknowledging local and Indigenous contributors is an ethical responsibility rather than a discretionary courtesy; ensuring visibility for all partners is vital to preventing the transformation of collaboration into exploitation.

Benefit sharing should be determined through community feedback, not assumed by researchers. For example, while researchers may offer training or materials, communities may prioritise ecological restoration, heritage protection, or advocacy support. Long-term partnerships—beyond the life of the grant or project—are important to fulfilling this principle. Benefit sharing could also be formalised through Memorandums of Understanding (MOUs) or reciprocal agreements that codify expectations for co-authorship, knowledge ownership, and financial distribution, thus institutionalising reciprocity beyond goodwill (Cochran et al. 2013; Bowrey et al. 2024). As communities across the Himalayas navigate unprecedented climate-induced shifts, it is ethically imperative for researchers to prioritise the lived experiences of residents and migrants, centring local voices in the discourse on mountain resilience.

Community-led monitoring should be actively supported through the development of collaborative partnerships that recognise and reinforce local authority (Malik and Ford 2025a). This process must involve two-way capacity sharing, enabling both researchers and community members to contribute knowledge, exchange skills, and co-create tools that enhance mutual learning and long-term stewardship. Researchers must be transparent about funding sources, intended outcomes, and institutional affiliations. This transparency builds trust and helps avoid the perception (or reality) of ulterior motives or neo-colonial interventions. In the context of historical knowledge extraction, reciprocity may also entail a reparative function, requiring researchers and institutions to dedicate resources to heal historical breaches of trust and return appropriated intellectual property or benefits (Tuck and Yang 2012).

An example to illustrate the benefit sharing is the case of the Himalayan rivers. The Himalayan rivers are sites of intense ecological pressure and political contestation (Davis 2023a). Large-scale hydropower projects, dam construction, and river-linking schemes—often initiated by state institutions without meaningful local engagement—have disrupted aquatic ecosystems, displaced communities, and ignited cross-border tensions (Manhas and Yadav 2024; Manna 2022; Rai 2024). Rivers such as the Teesta and Subansiri have become flashpoints of conflict between development goals, ecological integrity, and Indigenous rights (Sahu 2020; Noolkar-Oak 2017). In these contested sites, benefit-sharing must move beyond transactional compensations toward support for community-led advocacy and legal recognition. For example, research can provide the independent evidence base necessary for communities to assert their rights to territorial integrity and ecological stewardship. By aligning research outputs with community-defined needs—such as documentation for environmental impact challenges or the revitalisation of river-based cultural practices—benefit-sharing becomes a tool for resisting ecological dispossession rather than a passive byproduct of data collection.

Ethical research engagement in contested Himalayan sites must therefore involve community-led benefit identification and avoid contributing to state or corporate agendas that exacerbate ecological dispossession. Benefits must be evaluated not just in economic terms, but through the lens of cultural revitalisation, territorial integrity, and ecological stewardship.

Given this layered context, ethical research in the Himalayas must be attentive to both the fragility of its highland ecosystems and the resilience of its culturally diverse communities. It must engage not just with the physical landscape, but with the cultural, political, and historical terrains that shape how knowledge is produced, circulated, contested, and benefitted in the region.

### Long-term commitment and accountability

The final principle centres on temporal and relational ethics—the recognition that ethical research relationships do not end with publication, and accountability does not end with data collection (Kisselburgh and Beever 2022; Cornish et al. 2023). Ethical research entails long-term commitment, accountability, and relational continuity. In the Himalayan context, where trust is built slowly and values are embedded in kinship, hospitality, and cultural life (Das 2024; Shrestha et al. 2024), this is particularly important. Long-term commitment is not a gesture of goodwill but a necessary ethical stance.

Researchers must remain available for community feedback, provide updates in local languages, and participate in post-research engagements, such as festivals, knowledge-sharing events, or advisory roles. This includes translating findings into accessible formats, remaining open to community critique, and co-developing future research questions that respond to evolving community concerns. This requires planning for post-research engagement at the grant application stage, budgeting for return visits, translations, or collaborative dissemination. Researchers should also facilitate access to publications and ensure that results are not misused by external actors, including governments, corporations, or NGOs. Ethical research must result in community-held knowledge products, not only academic outputs. This could include story maps, policy briefs, school materials, or digital archives accessible within the region.

Institutional ethics protocols often fail to address what happens after fieldwork (Schmidt 2021; Poopuu and Van den Berg 2021). By contrast, Indigenous ethics stress that relational responsibility does not expire (Koggel et al. 2024; Tynan 2021). This temporal commitment should be viewed not merely as a personal virtue, but as a methodological necessity that mitigates the ‘hit-and-run’ research cycles common in global scholarship (Durán del Fierro 2023; Deranger et al. 2022; Hill et al. 2023). Researchers must remain accountable not only to their academic institutions but to the people and places that hosted them.

Mechanisms of accountability may include community ethics audits, public presentations, or co-authored policy briefs. Researchers must be willing to make reparations if harm was caused and contribute to the community’s ongoing wellbeing. This demand for perpetual accountability is a cornerstone of decolonial praxis, recognising that the historical and material consequences of research are ongoing (Fanon 1963).

As Marie Battiste (2016) and Linda Tuhiwai Smith (2021) argue, research must be *for* and *with* Indigenous and local communities—not merely ‘about’ them. In the Himalayas, long-term engagement may also entail researchers being called back for guidance, teaching, or advocacy, roles that require humility, listening, and readiness to serve in new capacities over time. In this sense, ethical research becomes a long-term relationship—a covenant rather than a contract—built on reciprocity, shared purpose, and mutual accountability.

The institutionalisation of accountability is important by demanding that universities and funding bodies allocate resources specifically for long-term data maintenance, post-project engagement, and community oversight, ensuring that the burden of continuity does not fall solely on the individual researcher (David-Chavez and Gavin 2018; Banks et al. 2016). Such accountability cannot be achieved through institutional ethics boards alone. It requires personal and political commitment to relational responsibility, grounded in mutual care, respect, and solidarity. This commitment aims to establish a reparative model of research that seeks to heal the historical rupture between academic practice and local sovereignty.

## Cross-disciplinary necessity of ethical principles

While the six principles proposed in this research are articulated through the lens of decolonial and relational ethics, their necessity extends across all disciplines, including the STEM, natural sciences, conservation biology, and technology development that operate in the Himalayas. The traditional separation between ‘hard’ and ‘soft’ sciences fails when technological and environmental interventions carry profound political, ethical, and human rights consequences. The rapid adoption of ecosurveillance and new digital tools in fields like conservation science—including camera traps, drones, ecological sensors, and artificial intelligence technology—necessitates adherence to a robust ethical framework, as these technologies often introduce fresh ethical and political issues that affect human populations (Young et al. 2022; Wong et al. 2025; Millner et al. 2024; Sarkar and Chapman 2021).

For instance, technologies intended for ecological monitoring can inadvertently or deliberately function as tools of surveillance and control, particularly in areas of long-standing conflict and geopolitical tension, blurring the line between conservation and coercion (Millner et al. 2024). Research has demonstrated the profound gendered impacts of this technological encroachment in the region, showing how surveillance technologies in conservation areas, such as the Corbett Tiger Reserve in India, can be used to intimidate, spy on, or restrict the essential livelihood activities of local women (Simlai and Sandbrook 2025). Such practices fundamentally violate principles of FPIC and expose marginalised communities to harm, suggesting that conservation practice remains gender blind or views local practices through a purely transactional lens.

Research in glaciology and environmental monitoring in the Himalayas (and more broadly in the cryosphere) also demands scrutiny. The collection of sensitive data on water resources, glaciers, hazard prediction (e.g., GLOFs), and ice loss can have profound socioeconomic and geopolitical implications, particularly in transboundary river basins (Dubey et al. 2024; Waseem and Chaudhry 2024). Beyond policy impacts, the dissemination of such findings requires a high degree of ethical care; labelling local environments as ‘hazardous’ can cause significant psychological distress and undue anxiety within downstream communities. Ethical engagement therefore necessitates that risk communication is co-developed with community members and local leaders, ensuring that data sharing is accompanied by actionable mitigation strategies and support systems rather than merely presenting a frightening characterisation of their landscape.

The placement of physical infrastructure—such as sensors, weather stations, or deep-drilling equipment on glaciers—can itself be viewed as a form of territorial claim or environmental disturbance and could be perceived as surveillance tools by communities especially in politically sensitive places. This requires transparency, rigorous FPIC, and long-term accountability, especially when working on mountains and other landscapes considered ecologically and culturally important by local communities.

Research across the physical sciences—ranging from geology and climatology to glaciology—often involves extractive sampling and the collection of sensitive data related to resource governance or conflict zones, requiring the same rigorous standards of benefit sharing and data sovereignty as social research (Whyte 2018; Gabrys 2016). Camera traps and drones used in forests and conservation areas are co-opted by state actors for intrusive monitoring, generating fear and disrupting traditional land-use practices, and transforming societal relationships with non-human worlds (Gabrys et al. 2025; Luque-Ayala et al. 2024; Sarkar and Chapman 2021). These technologies—though framed as value-neutral or ecologically necessary—have real-world socio-political impacts, reinforcing power imbalances and reducing communities to passive subjects of observation (Omodan 2025b; Rocco 2025; Wong et al. 2025). Such practices reflect a form of ‘green surveillance’ that aligns with broader patterns of settler environmentalism, where conservation is enacted through exclusion and discipline. The misapplication of environmental technologies illustrates that ethical engagement cannot be decoupled from natural sciences.

Therefore, these six ethical principles—spanning relationality, consent, collaboration, data sovereignty, and long-term accountability—are essential for all scientists, regardless of discipline, to ensure that technological and scientific advancements serve community needs and local sovereignty rather than reproducing colonial, statist, and gendered asymmetries of power. Ethical research thus demands an interdisciplinary commitment to respect, relationality, and reciprocity across all domains of inquiry, ensuring that knowledge production serves community benefits and long-term ecological well-being.

## Challenges and considerations

While the ethical principles proposed in this paper contribute to the ongoing effort to operationalise decolonial research in the Himalayas, their implementation is not without complexity. Researchers must contend with institutional, geopolitical, and logistical challenges that may hinder the application of these frameworks. Implementing these principles is neither easy nor straightforward. Researchers often face institutional pressures—tight timelines, rigid funding frameworks, and disciplinary publication demands—that disincentivise deep community engagement (Bell and Lewis 2023; Giannelos et al. 2024). State-imposed research restrictions, particularly in politically sensitive zones can make long-term collaboration risky or impossible. Communities themselves are not monolithic (Chakraborty et al. 2021). Power hierarchies within communities can shape who speaks, who is heard, and who is erased, thus complicating who ‘the community’ is. In many Himalayan settings, local leadership and knowledge validation are often dominated by male elders or religious elites, marginalising women, youth, lower-caste groups, and the landless (Chakraborty 2018; Yadav 2017; Imtiaz 2022; Kapadia 2017). Even within what may be formally labelled a ‘community,’ access to knowledge production, consent processes, and benefit-sharing may be unequally distributed. As such, any attempt to engage ‘the community’ must critically interrogate who is being represented, who is left out, and how internal power relations shape participation and silence. This internal stratification complicates the notion of ‘community’ as a unified entity (Scott 2014) and demands that researchers remain attentive to intra-community exclusions when engaging in participatory or ethical research. Ethical research must therefore be intersectional, attuned to internal as well as external forms of marginalisation.

I outline key obstacles and dilemmas, providing a nuanced perspective on the practicalities of ethical research.

### Ontological bias and epistemic privilege

An important challenge to ethical research in the Himalayas lies in the persistence of ontological bias—the assumption that the world can only be known through certain dominant categories, such as objectivity, positivism, measurement, universality, or mechanistic processes (Valore 2024; Vaditya 2018; Chakraborty et al. 2019). This bias often renders non-Western, Indigenous, or community-based realities, known as the pluriverse, as illegible, irrational, or subordinate (Kuokkanen 2011; Escobar 2018; Blaser and De la Cadena 2018). As a result, Indigenous or community-based ontologies—such as cultural understandings of Himalayan mountains, the personhood of glaciers, or relational kinship with water—are systematically dismissed or reduced to mere ‘local knowledge’ to be mined for data, rather than respected as a comprehensive system of reality, resulting in the othering of these knowledge systems (Smith 2021; Agrawal 1995). This reductionist approach fundamentally damages the relationship between the researcher and the community, violating the principle of relationality (Principle 1)

As a result, a research question regarding water security is stripped of its socio-cultural context. For example, a hydrologist may treat a mountain as purely an accumulation of ice mass and rock, ignoring its community-defined importance. This ontological biasness prevents the meaningful FPIC (Principle 2) required for physical interventions (e.g., placing sensors) and undermines Principle 1 (relationality), which demands engaging with equitable relationships with communities (Jennings et al. 2025; Brant et al. 2023).

This structural bias is reinforced by epistemic privilege, where academic and funding institutions implicitly claim the sole authority to validate knowledge (Fricker 2007). This privilege dictates which questions are ‘scientific,’ which methods are ‘rigorous,’ and who is permitted to hold authorship (Principle 3). This bias is amplified by epistemic privilege, where institutional power grants external researchers the right to define the research question, methodologies, and final interpretation. This is evident when GLOF hazards are framed through purely hydrological models, isolating the crisis to physical parameters quantified via remote sensing, empirical volume estimates, and hydrodynamic numerical simulations (e.g., HEC-RAS) of potential breach discharge and downstream inundation area (Maurer et al. 2020; Veh et al. 2019; Rawat et al. 2023; Allen et al. 2016; Lala et al. 2017; Cook et al. 2018). There is often an unconscious epistemic bias toward technical datasets, which are viewed as more ‘robust’ within dominant research models. However, this prioritisation can systematically obscure situated community concerns, resulting in a reductionist understanding that ignores the social, economic, and political drivers of the crisis. It risks marginalising the lived experiences and intergenerational knowledge of local communities, effectively decoupling the physical phenomenon from its profound socio-political context.

This privilege, by prioritising publication and metric-driven success over community needs, reinforces colonial knowledge asymmetries (Fricker 2007) and the very structures the six principles proposed here seek to dismantle. For Himalayan communities, this means their deep, situated, intergenerational knowledge, which may be expressed through storytelling or ritual, is often subordinate to knowledge expressed through peer-reviewed articles, quantitative datasets, or satellite imagery. The cumulative effect of these obstacles is the reproduction of colonial knowledge hierarchies, wherein research serves to extract and interpret information for external benefit, rather than to empower the community’s own self-determination (Smith 2021).

Overcoming these obstacles requires a fundamental shift beyond protocol to embrace epistemic pluralism. This mandates radical epistemic humility on the part of the researchers, requiring the acknowledgement of non-Western ontologies and the active surrender of control over the knowledge validation process to local partners. Importantly, this commitment must extend to making all knowledge products—including technical models, data, and findings—accessible in non-technical formats that are easily understood and usable by the communities they concern.

### Institutional constraints and bureaucratic ethics

Academic institutions often impose rigid ethical review frameworks—typically designed within Euro-American ethical paradigms (Sherman et al. 2012; Hou 2025; Sugiharto 2024)—which may not align with the relational and context-sensitive approaches needed for ethical work with Indigenous and local Himalayan communities. Institutional Review Boards (IRBs) typically focus on individual consent, anonymity, and risk minimisation, but sometimes overlook cultural protocols, collective consent processes, or Indigenous worldviews of relationality and responsibility (Kuhn et al. 2024; Appiah et al. 2024). IRBs rarely recognise community governance structures such as village or community councils as legitimate ethical arbiters (Fielding-Miller et al. 2022; Su 2022). This can create friction between institutional ethics requirements and community-led accountability mechanisms. In some cases, researchers are forced to navigate dual ethical systems, which may conflict in process or expectation.

A further complexity lies in the internal politics of communities themselves. While participatory and collaborative research frameworks aim to decentralise authority, they may inadvertently rely on local elites or gatekeepers—such as male elders, religious figures, or state-aligned actors—who do not represent the full spectrum of community perspectives. In many Himalayan contexts, Dalit, tribal, landless, or unmarried women are structurally excluded from village councils or ritual decision-making (Verma and Khadka 2016; Pyakurel 2021; Rathee 2024; Baral et al. 2024). Ethical research must therefore engage with intersectionality as an analytic and methodological tool, actively seeking out marginal voices and being reflexive about how representation is negotiated and contested within communities. This requires working across intra-community tensions and being prepared to navigate disagreement, refusal, or ambiguity.

### Funding limitations and temporal pressures

Funding cycles are often short-term and project-based, creating a fundamental temporal misalignment with the long-term commitment required for ethical, reciprocal research (Wicher and Frankus 2024; Gallagher et al. 2024). Contextual knowledge sometimes presents a challenge for the co-development of research aims as authentic co-design requires a pre-existing depth of understanding that rarely fits within standard grant timelines. Building trust, facilitating community participation, co-producing knowledge, and ensuring dissemination in accessible formats demand significant time and resources—far beyond typical research grant timelines. Budgetary constraints may limit the ability to offer fair compensation, hire local co-researchers, or translate materials into local languages. Without adequate support, even well-intentioned researchers may fall into extractive practices, despite their ethical commitments.

Several core tensions that complicate the implementation of ethical research principles are: *How can researchers co-develop a research proposal without funding to spend (a long) time in the field beforehand? How can researchers ensure that ‘the community’ questions are those that are interesting to a funder? How can researchers involve people in data interpretation and publications when faced with barriers to formal literacy, or—more complexly—when local knowledge systems and worldviews directly contest the scientific findings being produced? How can researchers change financial regulations within the institution to enable informal payments for peoples’ time/services? How can researchers sustain a long-term commitment to the people they are working with when the funding stops after, say, 3 years or so?*

Overcoming this requires funders to adopt longer, more flexible funding models tailored for relationship-building, and for institutions to prioritise and mandate reciprocal budgetary allocations for community-driven priorities.

### Geopolitical sensitivities and access restrictions

The Himalayan region spans multiple countries, each with distinct political contexts, border tensions, and research access regulations (Davis et al. 2021; Patel 2023). In politically sensitive areas, state surveillance, military presence, and nationalist agendas may shape who is allowed to research, what topics are permissible, and how findings can be disseminated (Pachankis 2022; Davis 2023b; Manna 2022). Ethnographic or environmental research may be seen as politically sensitive, especially when it involves cross-border affiliations, cultural practices, or contested ecological zones. Navigating these dynamics without compromising community safety or autonomy is a complex ethical task. Researchers are therefore ethically obliged to prioritise participant security and avoid methodologies that inadvertently expose communities to state scrutiny, geopolitical conflict, or legal repercussion.

### Cultural and linguistic plurality

The Himalayas are not a monolith; the region is characterised by vast cultural diversity (Sati 2023; Chakraborty 2021). A principle or process that is ethical in one community (e.g., a Buddhist village in Mustang) may not be appropriate or meaningful in another (e.g., a tribal village in Arunachal Pradesh). This plurality requires hyper-contextualised research ethics, attuned not only to language differences but to cosmological, gender, age, and caste-based variations in power and voice. Ethical practice, therefore, cannot rely on one-size-fits-all protocols but must be flexible and dialogic in nature.

### Power imbalances within communities

While decolonial research emphasises community empowerment, researchers must remain aware of intra-community power hierarchies (Malherben et al. 2022; Gallardo Lastra et al. 2024; Maher and Loncopán 2024). Gender, caste, age, class, or religious status may shape who gets to speak, who is heard, and who is silenced. Consulting only local elites or gatekeepers may reproduce internal inequalities, undermining the participatory intentions of the research. Ethical engagement thus demands an intersectional lens, actively seeking marginalised voices within the community and being reflexive about the researcher’s own positionality in shaping relationships and interpretations.

### Researcher positionality and reflexivity

Researchers themselves—especially those from outside the community or region—must constantly interrogate their own positionality, privilege, and epistemological assumptions (Secules et al. 2021; Kamlongera 2023). Even with participatory frameworks, dynamics of power, language, education, and institutional backing shape interactions (Blanco et al. 2022; Tesfaye et al. 2025). Ethical research requires not only structural changes but also inner work: humility, self-questioning, and a willingness to decentre oneself. This involves being open to critique, prepared to abandon or revise projects, and committed to accountability beyond institutional expectations.

## Conclusion

The Himalayan region is shaped by cultural depth and geopolitical tension, so research cannot be a neutral or purely intellectual activity. It is always political, always relational, and always embedded in power. This paper argues that ethical research in the Himalayas must begin from a recognition of these entanglements and proceed with humility, reflexivity, and shared commitment. The ethical challenges of conducting research in the Himalayas are not simply procedural—they are deeply embedded in the histories, politics, and power asymmetries that shape who gets to produce knowledge, under what conditions, and for whose benefit. This paper argues that conventional research models, often rooted in Eurocentric, extractive, and institutional paradigms, are fundamentally inadequate for engaging with the cultural and ecological richness of the Himalayan region. Instead, what is required is a decolonial ethical framework grounded in relational accountability, knowledge co-production, and the sovereignty of Indigenous and local communities. The six ethical principles proposed here are intended to guide researchers away from extractive habits and toward collective, community-affirming, ecologically attuned research relationships, and offer a roadmap for more respectful, inclusive, and reciprocal research engagements. These principles are not intended to be universally prescriptive, but rather adaptable guides that must be contextualised within the specific cultural, ecological, and political realities of each Himalayan community.

While these principles are grounded in the specific cultural and ecological contexts of the Himalayas, they are not geographically bounded. Rather, they offer a trans-regional framework applicable to other diverse landscapes—such as the Andes, the Amazon, or Sub-Saharan Africa—where research must navigate similar legacies of extractivism. While the substance of the principles is universal, their operationalisation must be meticulously calibrated to the unique political and social realities of each specific community.

Importantly, these principles challenge researchers to move beyond bureaucratic ethics and toward a practice of humility, reflexivity, and long-term solidarity. Ethical research is not an abstract checklist but a lived relationship—one that must be nourished with trust, mutual respect, and shared vision. For institutions and funders, this means rethinking timelines, evaluation criteria, internal processes, and modes of dissemination. For researchers, it demands a shift in positionality—from expert to collaborator, and from extractive observer to ethical participant.

The Himalayas are not merely sites of knowledge to be mined; they are homes, cultural landscapes, and living repositories of diverse worldviews. Research conducted in these regions must not replicate the violences of colonial knowledge production, but instead contribute to epistemic justice, community empowerment, and intergenerational resilience. The future of ethical research in the Himalayas lies not in expanding access to data, but in deepening commitment to relationship, care, and co-existence. Researchers must ask not only *‘what can we learn from the Himalayas?’* but also *‘how can our work be of service to the people and places we learn from?’* Only then can research become a truly ethical, decolonial, and transformative practice.

## Data Availability

All data is available within the manuscript.
